# The Influence of the Osmotic Dehydration Process on Physicochemical Properties of Osmotic Solution

**DOI:** 10.3390/molecules22122246

**Published:** 2017-12-16

**Authors:** Krzysztof Lech, Anna Michalska, Aneta Wojdyło, Paulina Nowicka, Adam Figiel

**Affiliations:** 1 Institute of Agricultural Engineering, Wroclaw University of Environmental and Life Sciences, 51-630 Wroclaw, Poland; adam.figiel@upwr.edu.pl; 2 Department of Fruit, Vegetable and Plant Nutraceutical Technology, Wroclaw University of Environmental and Life Sciences, 51-630 Wroclaw, Poland; anna.michalska@upwr.edu.pl (A.M.); aneta.wojdylo@upwr.edu.pl (A.W.); paulina.nowicka@upwr.edu.pl (P.N.)

**Keywords:** *Aronia melanocarpa* L., chokeberry juice, filtration, osmotic dehydration, antioxidant capacity, physical properties, carrot and zucchini

## Abstract

The osmotic dehydration (OD) process consists of the removal of water from a material during which the solids from the osmotic solution are transported to the material by osmosis. This process is commonly performed in sucrose and salt solutions. Taking into account that a relatively high consumption of those substances might have a negative effect on human health, attempts have been made to search for alternatives that can be used for osmotic dehydration. One of these is an application of chokeberry juice with proven beneficial properties to human health. This study aimed to evaluate the physicochemical properties of the OD solution (chokeberry juice concentrate) before and after the osmotic dehydration of carrot and zucchini. The total polyphenolics content, antioxidant capacity (ABTS, FRAP), dynamic viscosity, density, and water activity were examined in relation to the juice concentration used for the osmotic solution before and after the OD process. During the osmotic dehydration process, the concentration of the chokeberry juice decreased. Compounds with lower molecular weight and lower antioxidant capacity present in concentrated chokeberry juice had a stronger influence on the exchange of compounds during the OD process in carrot and zucchini. The water activity of the osmotic solution increased after the osmotic dehydration process. It was concluded that the osmotic solution after the OD process might be successfully re-used as a product with high quality for i.e. juice production.

## 1. Introduction

The osmotic dehydration (OD) process consists of the removal of water from fresh material (i.e. fruits and vegetables) wherein the solutes from the osmotic solution are transported to the plant material [1]. OD is a pre-treatment process, which has many advantages such as improving the physical and chemical food properties including colour, flavour, aroma, and texture, as well as possibly improve the biological activity of the products [2]. This process prolongs the storage time of the products, requires a shorter drying time, and is energy efficient when compared to the other drying techniques [1]. OD is usually followed by i.e., conventional drying due to the relatively high moisture content in OD products [3,4,5]. The OD process is commonly performed in sucrose and salt solutions [6,7]; however it might also be conducted in sorbitol [8], corn syrup [9], and starch syrup [10]. Taking into account that a relatively high consumption of those substances might have a negative effect on human health, numerous attempts have been made to search for alternatives [11,12]. One such alternative is the application of fruit and vegetable juice concentrates e.g. chokeberry [13], pomegranate and apple [14], sour cherries [15], blackcurrant, raspberry, quince [16], and grape [17], with proven beneficial properties. The ratio of material to solution during osmotic dehydration is 1:3 *w*/*w* [13], 1:4 *w*/*w* [10], 1:5 *w*/*w* [18], and 1:10 *w*/*w* [3]. For practical purposes, the sample:solution ratio ca. 1:3 is considered as an optimum [19]. 

During the OD process the attention is focused on the OD products, while the OD solution is regarded as a waste [20]. When fruit and vegetable concentrated juices were used as hypertonic solutions applied for the OD process, their properties were omitted [13,14,21]. During the OD process, solutions that contain numerous biologically active components significantly influence the quality of the final products [14,15]. Solutes that are moved into the material subjected to the OD process from the hypertonic solutions caused a decrease in their content in the OD solution. Simultaneously, the water that is removed from the material subjected to the OD process is transferred into the OD solution, resulting in a dilution of the solution. This was observed in the case of cherries [16]. The osmotic solution might be used several times, even up to five times, taking into account the appropriate ratio between the material and the OD solution of 1:10 (*w*/*w*) [22]. Due to the fact that the OD process is basically performed at 30 °C (up to 50 °C) over 4 h, the solution is prone to microbiological contamination [23], thus the quality of the OD solution should be controlled. What is more, during the OD process the solid gain from the materials is transferred to the OD solution, thus enriching its composition [24]. Taking into account that fruit and vegetable juice are more expensive than the salt or sucrose solutions applied for the OD process, their re-use would be a practical application.

Therefore, the aim of this study was to evaluate the physicochemical properties of the chokeberry juice concentrate used as an OD solution before and after the osmotic dehydration process.

## 2. Results and Discussion

### 2.1. Chemical Composition of Filtrated Chokeberry Juice

In order to examine the chemical composition of chokeberry juice used as an osmotic agent, the polyphenolics content in different juice fractions was evaluated, and the results are provided in Table 1. Their content in non-filtrated juice was similar to that obtained by Nowicka et al. [16]. The filtration resulted in changes in the content of polyphenols. In general, the filtration of chokeberry juice by filters with a pore size as small as 1.2 μm resulted in a slight increase in the polyphenols content, whereas further filtration (below 1.2 μm) diminished their content in the juice. suggesting the presence of low molecular weight compounds in such fractions. Thus, the presence of the different weight compounds in the osmotic solution might have a significant influence on the osmotic dehydration process, especially when the non-uniform structure of plant materials is concerned. What is more, the content of polyphenolic compounds is correlated with the antioxidant capacity. It can be observed that the antioxidant capacity values slightly increase for the juice fraction obtained after filtration by 1.2 μm pore size filters (Table 1), regardless of the method applied. Thus, the polyphenolic compounds present in a chokeberry juice might play a role in the products′ final properties. 

Going into detail, the composition of polyphenolic compounds in chokeberry juice is presented in Table 2. Similar to the findings of Nowicka et al. [16], among the compounds identified, the highest content of polymeric procyanidins was indicated, followed by chlorogenic, neochlorogenic, and quercetin-3-glucoside contents. Application of the different size filter membranes has a strong impact on the content of the polyphenolic compounds with a relatively high molecular weight, i.e., polymeric procyanidins. It was observed that their content slightly increased when the size of membrane pores was equal to or greater than 1.2 μm. When the membrane with a pore size below 1.2 μm was applied, the content of polymeric procyanidins in chokeberry juice diminished. It can be concluded that different pore sizes in the plant materials subjected to the osmotic dehydration process can have a significant influence on their distribution through the plant cells. What is more, the transfer of the polyphenolic compounds into the plant material might be selective due to the molecular weight. 

### 2.2. Osmotic Dehydration (OD) of Carrot and Zucchini

**Figure 1:**
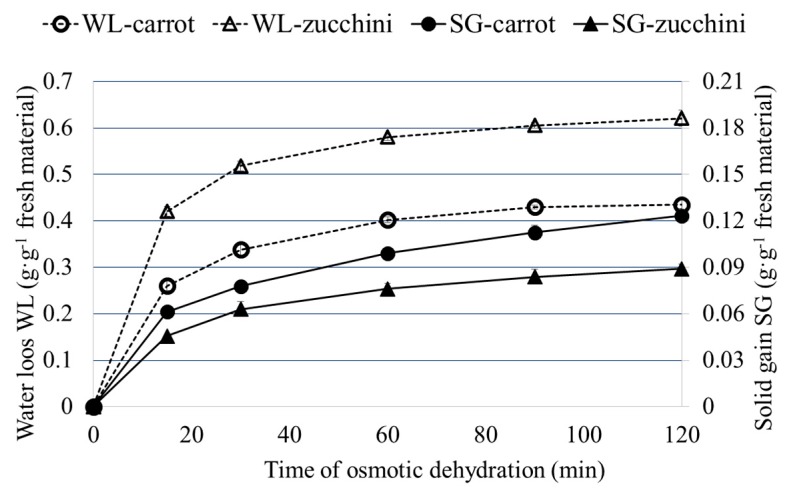
Water loss (WL) and solid gain (SG) during the osmotic dehydration of carrot and zucchini.

Figure 1 shows water loss (WL) and solids gain (SG) during the osmotic dehydration of carrots and zucchini in concentrated chokeberry juice. At the beginning of the process, the exchange of mass between the raw material and the solution is very intensive, but over time the intensity of the mass transfer decreases asymptotically. This behavior was observed during the dehydration of cherry fruits in concentrated apple juice [15]. WL for zucchini was higher than that for carrots because of its significantly higher initial moisture content, which was 19.12 kg·kg^−1^db for zucchini and 9.31 kg∙kg^−1^db for carrots. However, the higher values of WL were accompanied by lower values of SG due to the large flow of water from the zucchini to the osmotic solution, which could hinder the transfer of solids from the osmotic solution to the zucchini. A similar relationship was obtained during the dehydration of papaya samples which differed in maturity and initial water content [25].

**Figure 2:**
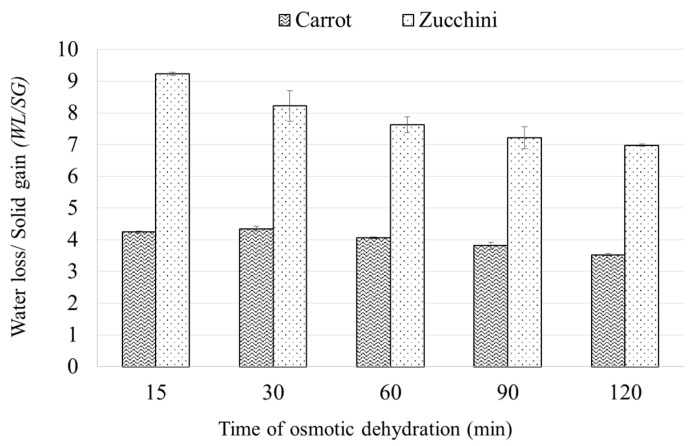
The ratio of water loss to solids gain (WL/SG) during the osmotic dehydration of carrot and zucchini.

Figure 2 represents the ratio of water loss to solids gain (WL/SG) during the dehydration of carrots and zucchini in concentrated chokeberry juice. At the beginning of the OD process, the advantage of WL over SG for zucchini was nine times, and in the case of carrots only four times. At a later stage of the process, the values of WL/SG ratio decreased significantly for zucchini and only slightly for carrots. Similar results were obtained during the dehydration of acerola fruits in sucrose solution [26]. Osmotic pressure in the raw material decreases as the moisture content increases, as the osmotic pressure depends on the water activity which is associated with the moisture content of the material. On the other hand, the osmotic pressure of hypertonic solution increases together with its concentration [27]. For a given solution, the difference in osmotic pressure is higher when dipping a material with higher humidity (zucchini) than with lower humidity (carrots). The greater the osmotic pressure difference, the more intensive the mass exchange, and for a material with high water content the WL is much stronger than the SG during OD. The transfer of water from the material to the solution and the transfer of solids from the solution to the material occurs in the same path [24], which has limited capacity. As such, higher WL enforces lower SG.

**Figure 3:**
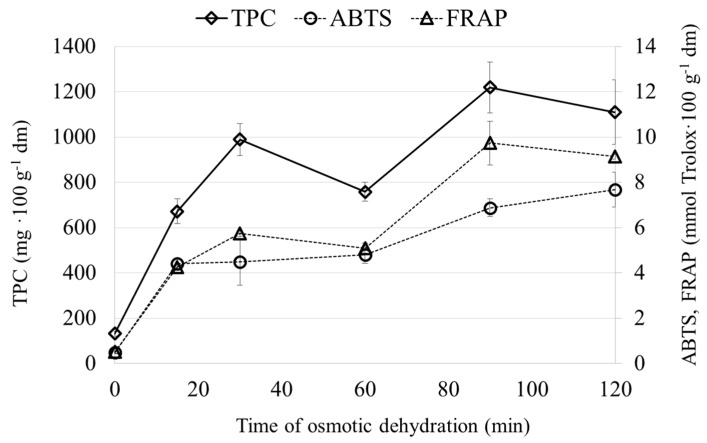
Chemical properties of carrots after osmotic dehydration in chokeberry juice.

**Figure 4:**
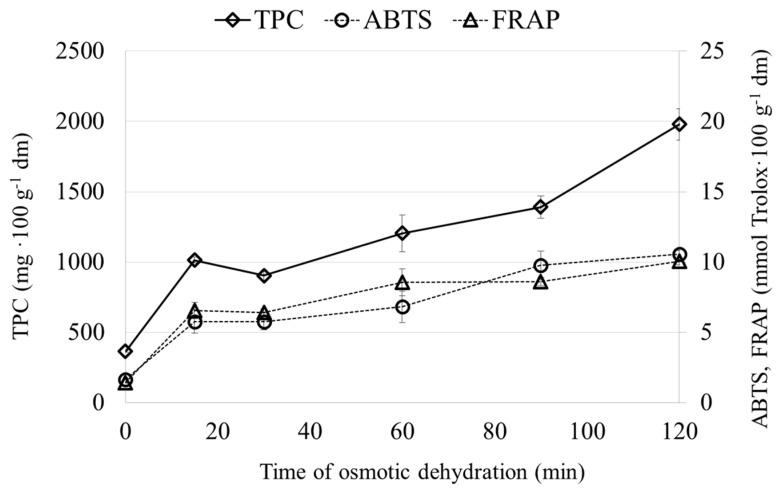
Chemical properties of zucchini after osmotic dehydration in chokeberry juice.

Figure 3 and Figure 4 showed the content of polyphenolics and the antioxidant capacity of the carrot (Figure 3) and zucchini (Figure 4) subjected to the OD process in a chokeberry juice concentrate. It was observed that the content of polyphenols significantly increased after OD. This was connected with the chemical composition of chokeberry juice and its antioxidant potential [28]. Similar results were obtained during the osmotic dehydration beetroot [13] and cherry [16] in chokeberry juice. It was noticed that the polyphenols content significantly increased in OD materials at the beginning of the process and, after 15 min for zucchini and after 30 min for carrot, their content was diminished, even the solid gain (SG) began to increase. Further processing resulted in an increase in the polyphenols content for both materials. A similar observation was made in the case of thawed cherry osmotically dehydrated in concentrated apple juice [15]. Such behavior, i.e., a decrease in polyphenols content with a simultaneous increase in solid gain, might be connected with the different sizes of particles present in concentrated chokeberry juice, which is dependent on their molecular weight (Table 1). At the beginning of the process, the osmotic solution enters the intercellular spaces between the cells that are relatively big in comparison to the plasmodesmata, whose diameter range from 20 nm up to 70 nm [29]. Thus, in the initial phase of the osmotic dehydration process, the particles from chokeberry juice enter the cells and smaller compounds take part in the exchange between the cell and the osmotic solution until plasmolysis occurs. After plasmolysis, cells lose their semi-permeable properties and the solution fills their its interiors [24]. The results presented in Table 1 confirm that the concentration of polyphenols in concentrated chokeberry juice changes after filtration. 

Similar to the polyphenols content, the antioxidant capacity—measured by the ability to scavenge the ABTS^+^ radical cations and by the FRAP method—increases approximately eight times in the case of carrot (Figure 3) and almost four times in the case of zucchini (Figure 4) at the beginning of the osmotic dehydration process. Similar results were obtained for model food composed of agar-agar gel osmotically dehydrated in salt and sucrose solutions with the addition of commercial grape seed extracts with relatively high antioxidant capacity [30]. From 30 up to 60 min of the OD process, the antioxidant capacity was at the comparable level, even though the solid gain increased. It was noticed that after 60 min of the process, the antioxidant capacity increased. It might be concluded that the antioxidant capacity is strictly connected to the polyphenols contents (Table 1).

### 2.3. Properties of Osmotic Solution (OS) after Osmotic Dehydration

Physical and chemical properties of the osmotic solution (chokeberry juice) after the osmotic dehydration of carrot and zucchini are provided in Table 3. During the osmotic dehydration process, the removal of water from the material to the solution and the migration of certain substances from the solution to the material result in the dilution of the osmotic solution. After 15 min, the concentration of the osmotic solution used for carrot dehydration was 36.2 °Brix and that for zucchini was at the level of 36.5 °Brix. Such a decrease in the concentration of the chokeberry juice was connected with a very intensive mass exchange noticed at the beginning of the process (Figure 1). During further dehydration (15 min up to 120 min), the solution concentration decreased much more slowly, reaching at the end of the process the concentration of 33.2 °Brix for carrots and 33.7 °Brix for zucchini. The lower Brix values after the osmotic dehydration process in comparison to the raw material was also noted in the case of blueberry dehydration in concentrated apple juice [31]. 

The content of polyphenols increased in the osmotic solution after the osmotic dehydration process. This might be connected with the selective transfer of the compounds present in the material as well as in the osmotic solution. 

During the osmotic dehydration process, the constituents from the osmotic solution enter the intracellular spaces and mass exchange takes place in the cells. The cell membrane is semi-permeable, and the size of the openings in the cellular system is probably smaller than the size of some particles in the solution [29]. Therefore, the larger particles responsible for the higher content of total polyphenols are likely to remain in the solution. This may cause an increase in their content in the solution after the OD process. However, a diminished content of polyphenols was reported during the dehydration of frozen cherries in concentrated fruit juices. This reduction was associated with a change in the structure of the cherries, which was destroyed by the removal of the stones and the freezing process [16]. In general, the antioxidant capacity (ABTS and FRAP) is strongly correlated with total polyphenols content [32]. After the OD process, the water activity of the OS increases due to the decrease of its concentration. A similar observation was made for concentrated apple juice [33]. The dilution process also contributed to the reduction in the density of the osmotic solution. The density of the concentrated juices was reduced due to the decrease in the concentration and the increase in the temperature [34]. Those parameters (concentration and density) might have an influence on the value of the osmotic pressure [35,36]. The viscosity of the OS significantly decreases at the beginning of osmotic dehydration process, depending on the temperature and concentration of the concentrated juice. What is more, viscosity varies exponentially with the concentration of concentrated juice; that is to say, the lower the concentration, the lower the viscosity [37]. 

## 3. Materials and Methods 

### 3.1. Materials

Commercial concentrated chokeberry juice was used in the experiment (Rauch Polska, Płońsk, Poland) (65 °Brix). The plant material used in the study was supplied by a local farm (Wrocław, Poland). It consisted of carrot (*cv*. Nerac) (Mc = 9.31 kg∙kg^−1^db) and zucchini (*cv*. Cora) (Mc = 19.12 kg∙kg^−1^db). Before the dehydration experiment, the material was washed and cut into slices (18 ± 0.1 mm in diameter, 3.35 ± 0.15 mm in thickness).

### 3.2. Filtration of Chokeberry Juice

Concentrated chokeberry juice was diluted to 20 °Brix. It was then filtered through a Cellulose Nitrate (CN) Membrane Filter (Sartorius AG, Goettingen, Germany) using membranes of smaller and smaller pore sizes. The pore sizes of the membrane filters were 8, 5, 3, 1.2, 0.8, 0.45, and 0.2 μm. After each filtration, chemical analyses were conducted. 

### 3.3. Osmotic Dehydration

The commercial concentrated chokeberry juice (40 °Brix) was used as an osmotic solution. The osmotic dehydration process of carrot and zucchini was performed in water baths at 45 °C for 90 min. The ratio of plant slices to osmotic solution was maintained at 1:3 (30 g:90 mL) and the mixture was manually agitated every 5 min [13,21].

Determination of mass transfer during osmotic dehydration (OD) required calculating weight reduction (WR), solid gain (SG), and water loss (WL) using following equations [15]:

(1) $$WR=\frac{w_{i}-w_{f}}{w_{i}}$$

(2) $$SG=\frac{s_{f}-s_{i}}{w_{i}}$$

(3) WL=WR+SG

where w_i_ and w_f_ are the initial and final (after OD) value for the weight of sample (g); s_i_ and s_f_ are the initial and final value for the solid content in the sample (g), respectively. WR, SG, and WL were presented as g∙g^−1^ fresh material.

### 3.4. Physical and Chemical Analyses 

#### 3.4.1. Moisture Content

The moisture content of materials was determined using a vacuum-dryer (SPT-200; ZEAMiL Horyzont, Krakow, Poland), where samples were kept at 70 °C at pressure 100 Pa for 24 h. The measurement was performed in triplicate and expressed as % of water. 

#### 3.4.2. Concentration of Chokeberry Juice

The concentration of chokeberry juice was measured using an Atago Digital Brix Refractometer, PAL-3 (Atago Co., Ltd., Tokyo, Japan). The measurement was performed in triplicate and expressed as °Brix.

#### 3.4.3. Water Activity (*a_w_*) of Chokeberry Juice

Water activity was measured for osmotic solutions before and after OD using a water activity meter AquaLab DewPoint 4TE (Decagon Devices Inc., Pullman, WA, USA) at 25 °C ± 0.5. The measurements were performed in triplicate. 

#### 3.4.4. Density of Chokeberry Juice 

The density was measured for osmotic solutions before and after OD. The density (ρ_t_) (kg∙m^−3^) of samples was determined as the ratio of the mass (m_j_) of the juice to the total volume of juice (V_j_) (Equation (4)):

(4) $$\rho_{t}=\frac{m_{j}}{V_{j}}$$

The juices were weighed with an analytical balance with an accuracy of 0.0001 g (XA 60/220/X Radwag, Radom, Poland), while the volume of juice was measured using a HumiPyc 2 Gas Pycnometer (InstruQuest Inc., Coconut Creek, FL, USA).

#### 3.4.5. Viscosity of Chokeberry Juice

Viscosity was measured for osmotic solutions before and after OD. The temperature of the osmotic solution was 45 °C. Viscosity was determined using a Vibro Viscometer SV-10 (A&D COMPANY, LIMITED, Tokyo, Japan)

### 3.5. Identification and Quantification of Polyphenols by the LC-PDA-MS Method 

The identification and quantification of polyphenols in chokeberry juice was prepared as described previously by Wojdyło et al. [38] and Nowicka et al. [16].

### 3.6. Antioxidant Capacity (TEAC ABTS and FRAP Methods)

The extracts were obtained by the sonication (2 × 10 min) of 500 mg of samples in 2 mL of 80% aqueous methanol. After being kept for 24 h at 4 °C in the dark, the extracts were centrifuged (1500× *g*; 10 min; 4 °C). The antioxidant capacity of extracts and chokeberry juice used as an osmotic agent was determined using Trolox Equivalent Antioxidant Capacity tests (TEAC ABTS) according to Re et al. [39]. The FRAP method was conducted in the abovementioned samples according to Benzie and Strain [40]. All measurements and analyses were performed in triplicate (*n* = 3) and the results were presented as an average mmol Trolox 100 g dm^−1^ (±SD).

### 3.7. Statistical Analysis

One-way analysis of variance (ANOVA) was performed by Statistica v.12.0 (StatSoft, Inc., Tulsa, CA, USA) in order to find out whether the differences in the averages were significant. Homogeneous groups were determined by the Tukey’s HSD test at a significance level of α = 0.05. 

## 4. Conclusions

The osmotic dehydration process in concentrated chokeberry juice significantly increased the polyphenols content and antioxidant capacity of the final product, i.e., carrot and zucchini. Besides the fact that during the OD process solids entered from the osmotic solution into the material, causing a decrease in its concentration, the polyphenols content and antioxidant capacity increased. This was due to the selective transport of the polyphenolics from the osmotic solution into the material. It was concluded that compounds with lower molecular weight and lower antioxidant capacity present in the concentrated chokeberry juice had a stronger influence on the exchange of compounds during the OD process in carrot and zucchini. Thus, the concentrated chokeberry juice used as an osmotic solution might be re-concentrated and re-used for further osmotic dehydration processes or might be applied as a semi-product in chokeberry juice production. The characterization of the osmotic solution led to the conclusion that there is a strong correlation between the concentration of concentrated chokeberry juice and its water activity, density, and viscosity. In short, the water activity in the osmotic solution increases, while the density and viscosity decrease with the decrease in juice concentration.

## Figures and Tables

**Table 1:** The content of polyphenols and antioxidant capacity in the chokeberry juice before and after filtration (*n* = 3).

Pore Sizes (μm)	Polyphenols (mg·100 g^−1^ dm)	TEAC ABTS (mmol Trolox·100 g^−1^ dm)	FRAP (mmol Trolox·100 g^−1^ dm)
no filtration	2919.4 ± 12.2 ^a,b,c,^*	26.74 ± 0.43 ^a,b^	21.33 ± 0.50 ^b,c^
8	3002.2 ± 133.9 ^a^	27.25 ± 0.30 ^b^	21.85 ± 0.06 ^c^
5	3081 ± 0.3 ^a^	26.48 ± 0.476 ^a,b^	21.14 ± 0.25 ^a,b^
3	3092.2 ± 24.4 ^a^	26.43 ± 0.41 ^a,b^	20.80 ± 0.19 ^a,d^
1.2	3116.5 ± 5.2 ^a^	26.53 ± 0.27 ^a,b^	21.34 ± 0.15 ^a,b,c^
0.8	2998 ± 85.9 ^a,b^	26.43 ± 0.30 ^a,b^	21.23 ± 0.73 ^a,b,c^
0.45	2811.5 ± 13.9 ^b,c^	25.98 ± 0.25 ^a^	20.74 ± 0.16 ^a,d^	20.74 ± 0.16 ^a,d^
0.2	2719.4 ± 7 ^c^	25.95 ± 0.35 ^a^	20.44 ± 0.48 ^d^

* Values followed by the same letter (^a,b,c,d^), within the same column, were not significantly different (*p* < 0.05) (Tukey’s HSD test).

**Table 2:** The content of identified polyphenolic compounds in chokeberry juice before and after filtration (mg·100 g^−1^ dm).

Pore Sizes (μm)	PolyMeric Procyanidins	Phenolic Acids			Flavonoids								
		Neochlorogenic Acid	*p*-Coumaric Acid	Chlorogenic Acid	Q-3-Rutinoside	Q-3-Galactoside	Q-3-Glucoside	Q-Arabinoside	Cya-3-Galactoside	Cya-3-Glucoside	Cya-3-Arabinoside	Cya-3-Xyloside	Derivatives of Cyanidin
no filtration	1857.4 ± 1.1 ^a,b,^*	251.2 ± 2.2 ^a^	9.22 ± 0.39 ^a^	400.1 ± 5.3 ^a^	28.16 ± 0.84 ^a^	18.52 ± 1.53 ^a^	66.68 ± 0.86 ^a,b^	28.61 ± 1.94 ^a^	1.69 ± 0.08 ^a^	164.5 ± 2.7 ^e^	9.73 ± 0.68 ^a,b^	74.4 ± 0.8 ^c^	9.24 ± 0.35 ^a,b^
8	1939.6 ± 134.3 ^a,b^	257.9 ± 8.7 ^a^	9.06 ± 0.41 ^a^	397.2 ± 1 ^a,b^	28.27 ± 0.86 ^a^	18.92 ± 0.14 ^a^	67.22 ± 2.1 ^a,b^	29.14 ± 0.4 ^a^	1.66 ± 0.06 ^a^	161.5 ± 3.3 ^c,e^	10.01 ± 0.5 ^a^	72.2 ± 2.2 ^b,c^	9.42 ± 0.2 ^a^
5	2029.6 ± 11.5 ^a^	263.8 ± 15.3 ^a^	8.67 ± 0.44 ^a^	393.7 ± 1 ^a,b^	27.81 ± 0.5 ^a^	18.74 ± 0.6 ^a^	66.48 ± 0.59 ^a,b^	28.92 ± 0.06 ^a^	1.77 ± 0.07 ^a^	153.8 ± 1.1 ^a,c^	9.43 ± 0.02 ^a,b^	68.9 ± 0.01 ^a,b^	9.32 ± 0.15 ^a,b^
3	2032.7 ± 22.8 ^a^	259.5 ± 0.3 ^a^	8.68 ± 0.06 ^a^	399.3 ± 1.1 ^a^	28.72 ± 0.04 ^a^	19.95 ± 0.02 ^a^	68.05 ± 0.3 ^a,b^	29.23 ± 0.07 ^a^	1.45 ± 0.01 ^a^	155.3 ± 0.1 ^a,c^	10.09 ± 0.15 ^a^	69.8 ± 0.3 ^a,b,c^	9.38 ± 0.48 ^a,b^
1.2	2048.1 ± 9.4 ^a^	270.6 ± 13 ^a^	9.09 ± 0.37 ^a^	401.4 ± 1.1 ^a^	28.78 ± 0.01 ^a^	19.62 ± 0.11 ^a^	67.84 ± 0.81 ^a,b^	28.9 ± 0.77 ^a^	1.59 ± 0.14 ^a^	152.9 ± 1 ^a,b^	9.69 ± 0.11 ^a,b^	68.8 ± 0.9 ^a,b^	9.06 ± 0.52 ^a,b^
0.8	1931.6 ± 116.1 ^a,b^	261 ± 7.3 ^a^	9.48 ± 0.24 ^a^	404.7 ± 12.9 ^a^	29.29 ± 0.5 ^a^	20.09 ± 1.24 ^a^	69.12 ± 1.28 ^b^	30.23 ± 1.68 ^a^	1.75 ± 0.14 ^a^	153 ± 3.2 ^a,b^	9.67 ± 0.08 ^a,b^	68.6 ± 1.8 ^a,b^	9.51 ± 0.1 ^a^
0.45	1770.9 ± 1.6 ^b^	263.1 ± 6.8 ^a^	9.34 ± 0.31 ^a^	395.3 ± 4.2 ^a,b^	27.91 ± 1.17 ^a^	19.66 ± 0.11 ^a^	67.12 ± 0.05 ^a,b^	28.24 ± 0.2 ^a^	1.69 ± 0.09 ^a^	145 ± 0.5 ^b,d^	9.12 ± 0.01 ^a,b^	65.1 ± 0.1 ^a,d^	9.03 ± 0.07 ^a,b^
0.2	1726.1 ± 5.6 ^b^	249.9 ± 9.4 ^a^	8.86 ± 0.01 ^a^	378 ± 0.6 ^a^	27.56 ± 0.1 ^a^	18.79 ± 0.49 ^a^	64.63 ± 0.48 ^a^	28.12 ± 0.24 ^a^	1.55 ± 0.03 ^a^	137.7 ± 2.2 ^d^	8.69 ± 0.04 ^b^	61.4 ± 1.3 ^d^	8.18 ± 0.21 ^b^

^#^ Q–quercetin; Cya–cyanidin; * Values followed by the same letter (^a,b,c,d,e^), within the same column, were not significantly different (*p* < 0.05) (Tukey’s HSD test).

**Table 3:** Physical and chemical properties in the osmotic solution (chokeberry juice) after the osmotic dehydration (OD) of carrots and zucchini.

Osmotic Solution	Time of Osmotic Dehydration (min)	Concentration of Osmotic Solution (° Brix)	Polyphenols TPC (mg GA·100 g^−1^ dm)	ABTS (mmol Trolox·100 g^−1^ dm)	FRAP (mmol Trolox·100 g^−1^ dm)	Water Activity (-)	Density (kg·m^−3^)	Viscosity (mPa·s)
Chokeberry juice after the OD of carrot	0	40 ± 0.1 ^e,^*	2909.4 ± 12.2 ^b^	26.95 ± 0.43 ^b^	21.54 ± 0.50 ^b^	0.944 ± 0.004 ^a^	1194.6 ± 24 ^a^	3.35 ± 0.1 ^c^
	15	36.2 ± 0.1 ^d^	3626.7 ± 77.1 ^a^	32.00 ± 0.36 ^a^	26.36 ± 0.49 ^a^	0.954 ± 0.006 ^a^	1174.3 ± 26.5 ^a^	2.9 ± 0.15 ^b^
	30	35.1 ± 0.1 ^c^	3596.1 ± 97.1 ^a^	30.91 ± 0.55 ^a^	25.50 ± 0.77 ^a^	0.956 ± 0.008 ^a^	1168.4 ± 31.2 ^a^	2.77 ± 0.18 ^a,b^
	60	34.4 ± 0.1 ^b^	3679.6 ± 100.3 ^a^	31.29 ± 1.01 ^a^	25.26 ± 0.61 ^a^	0.958 ± 0.009 ^a^	1164.5 ± 20.6 ^a^	2.67 ± 0.15 ^a,b^
	90	33.4 ± 0.1 ^a^	3619.1 ± 60 ^a^	30.98 ± 0.60 ^a^	25.96 ± 0.19 ^a^	0.96 ± 0.005 ^a^	1159.5 ± 16 ^a^	2.55 ± 0.09 ^a,d^
	120	33.2 ± 0.1 ^a^	3770.4 ± 75 ^a^	31.72 ± 0.54 ^a^	27.20 ± 1.79 ^a^	0.96 ± 0.006 ^a^	1158.1 ± 28.6 ^a^	2.52 ± 0.1 ^a^
Chokeberry juice after the OD of zucchini	0	40 ± 0.1 ^E,#^	2919.4 ± 12.2 ^C^	26.95 ± 0.43 ^D^	21.54 ± 0.60 ^A^	0.944 ± 0.004 ^A^	1194.6 ± 24 ^A^	3.35 ± 0.1 ^C^
	15	36.5 ± 0.1 ^D^	3548.8 ± 54.4 ^B^	31.45 ± 0.60 ^A,B^	22.13 ± 0.56 ^A,B^	0.953 ± 0.008 ^A^	1176 ± 20.5 ^A^	2.94 ± 0.12 ^B^
	30	35.3 ± 0.1 ^C^	3481.9 ± 65 ^A,B^	32.48 ± 0.41 ^B^	23.31 ± 0.54 ^B,C^	0.956 ± 0.006 ^A^	1169.6 ± 30.3 ^A^	2.8 ± 0.11 ^A,B^
	60	34.9 ± 0.1 ^B^	3486 ± 50.3 ^A,B^	31.35 ± 0.55 ^A,B^	23.59 ± 0.51 ^C^	0.957 ± 0.008 ^A^	1167.1 ± 32.5 ^A^	2.73 ± 0.13 ^A,B^
	90	33.8 ± 0.1 ^A^	3387.5 ± 24.9 ^A^	30.05 ± 0.76 ^A,C^	21.97 ± 0.10 ^A,B^	0.959 ± 0.006 ^A^	1161.6 ± 25.5 ^A^	2.6 ± 0.09 ^A^
	120	33.7 ± 0.1 ^A^	3474.9 ± 60 ^A,B^	28.63 ± 0.62 ^C^	21.45 ± 0.65 ^A^	0.959 ± 0.008 ^A^	1160.9 ± 20.2 ^A^	2.58 ± 0.11 ^A^

* Values followed by the same lowercase letters (^a∓e^), within the same column, were not significantly different (*p* < 0.05) (Tukey’s HSD test). ^#^ Values followed by the same capital letters (^A∓E^), within the same column, were not significantly different (*p* < 0.05) (Tukey’s HSD test).
